# Is there a relationship between cardiorespiratory fitness and executive performance as function of mental workload in young adults?

**DOI:** 10.3389/fpsyg.2022.932345

**Published:** 2022-07-22

**Authors:** Nounagnon Frutueux Agbangla, Marion Pater Maire, Pauline Maillot, Damien Vitiello

**Affiliations:** ^1^Laboratory URePSSS – SHERPAS (ULR 7369), Université d’Artois, Université du Littoral Côte d’Opale, Université de Lille, UFR STAPS, Liévin, France; ^2^Institut des Sciences du Sport-Santé de Paris (I3SP - URP 3625), Université Paris Cité, Paris, France

**Keywords:** cardiorespiratory fitness, oxygen uptake, executive functions, perceived difficulty, young adult

## Abstract

In the current study, we have decided to investigate the relationship between cardiorespiratory fitness and executive functions in young adults as a function of mental workload. To achieve our objectives, we have solicited 29 young adults (18–25 years; 12 women) who have first realized the Random Number Generation (RNG) task with two levels of complexity. After each level of complexity, the participants were asked to report on their perceived difficulty. Secondly, participants performed the RABIT^®^ test, during which oxygen consumption was measured through the Metamax 3B-R2. The results showed that executive performance and perceived difficulty deteriorate with increasing task complexity. Additionally, oxygen consumption increased significantly to reach a peak during the hardest phase of the RABIT^®^ test. Finally, as in previous studies, we could not observe a correlation between cardiorespiratory fitness and executive functions. Our findings support the lack of a direct relationship between cardiorespiratory fitness and executive functions. Future studies should explore the relationship between the composite measure of executive function, hemodynamic activity, and cardiorespiratory fitness in healthy youth and their peers with cardiovascular disease. This will examine an indirect effect of Cardiorespiratory fitness (CRF) on Executive functions (EFs) through brain activity.

## Introduction

Executive functions (EFs) are cognitive processes that regulate thought and action in unusual situations (Friedman et al., 2006). They can be subdivided into core EFs, namely inhibition, working memory, cognitive flexibility, or shifting, and higher-order EFs, such as reasoning, problem solving, and planning (Diamond, 2013). According to Diamond, inhibition—involves being able to control one’s attention, behavior, thoughts, and/or emotions to override a strong internal predisposition or external lure and instead do what’s more appropriate or need—(Diamond, 2013; p. 137). As regards working memory, it—involves holding information in mind and mentally working with it—(Diamond, 2013; p. 142). Lastly, cognitive flexibility involves two aspects. —One aspect is being able to change perspectives spatially. Another aspect involves changing how we think about something—(Diamond, 2013; p. 149).

Cardiorespiratory fitness (CRF) —reflects the integrated ability to transport oxygen from the atmosphere to the mitochondria to perform physical work— (Ross et al., 2016; p. 654). It can be measured directly using a maximal effort test that is performed on a treadmill or a cycloergometer (Hayes et al., 2013). Apart from a direct measurement, CRF can be estimated from the peak work rate achieved on a treadmill or a cycle ergometer or from non-exercised algorithms (Ross et al., 2016). The positive association between cardiorespiratory fitness (CRF) and executive performance is fairly well documented in the scientific literature (Huang et al., 2015; Pindus et al., 2015; Scott et al., 2016; Kawagoe et al., 2017; Mekari et al., 2019; Pentikäinen et al., 2019; Zhan et al., 2020; Cabral et al., 2021; Zhu et al., 2021). However, although many studies have highlighted this relationship in children, teenagers (Huang et al., 2015; Pindus et al., 2015; Zhan et al., 2020; Cabral et al., 2021; Zhu et al., 2021), and older adults with or without cognitive impairment (Kawagoe et al., 2017; Mekari et al., 2019; Pentikäinen et al., 2019), studies in young adults are scarce and there is result discrepancy between these studies (Hayes et al., 2016; Scott et al., 2016). Indeed, Scott et al. (2016) have highlighted the relationship between CRF and EFs (working memory, shifting, and planning). However, other authors were unable to show this relationship in young adults (Hayes et al., 2016). These discrepancies could be explained by methodological differences that relate to the sample and the executive tasks used to measure EFs. For example, Scott et al. (2016) enrolled 120 women and used different tasks (i.e., Modified flanker test, Go/No Go, Sternberg’s working memory, Tower of London task) to measure EFs, whereas Hayes et al. (2016) included 34 young adults including 18 women, using the Delis Kaplan Executive Function System task and calculated a composite executive function score. To sum up, it appears that very few studies have examined the relationship between CRF and EFs in young adults. In addition, a recent umbrella review of randomized controlled trials on the effects of physical exercise on cognition suggests that the evidence might not be reliable and therefore no strong conclusions should be drawn based on existing evidence (Ciria et al., 2022).

Mental workload is a multidimensional concept that depends on two following factors: (1) the demand of the task and (2) the cognitive resources available to the individual (Hardy and Wright, 2018). This concept is defined as the mental effort exerted to maintain the execution of a task at an acceptable level when the level of complexity of the task changes (Brookhuis and de Waard, 2010). Several studies have demonstrated the effect of mental workload on executive performance but also on brain activity during executive tasks with different mental workloads (Towse, 1998; Jahanshahi et al., 2006; Ayaz et al., 2012; Fishburn et al., 2014; Herff et al., 2014; Agbangla et al., 2019). But despite these effects of mental workload on executive performance and brain activity, several studies that have tested the relationship between executive performance and CRF have not modulated the mental workload of the executive task to examine its effect on the relationship between executive performance and CRF (Huang et al., 2015; Pindus et al., 2015; Hayes et al., 2016; Scott et al., 2016; Kawagoe et al., 2017; Pantzar et al., 2018; Mekari et al., 2019; Pentikäinen et al., 2019; Zhan et al., 2020; Cabral et al., 2021; Meijer et al., 2021; Zhu et al., 2021). Beyond the paucity of studies in young adults on this issue, few studies investigating the relationship between CRF in young adults did not consider the level of mental workload required by an executive task.

Based on the above observations, the present brief report aims to re-examine the relationship between CRF and EFs in young adults while considering the level of mental workload. To achieve this goal, we defined the complexity level of the task as follow: an executive task with two levels of mental workload (i.e., Random Number Generation). In addition, we measured the CRF with an effort test (i.e., RABIT^®^ test). Although our main objective was to re-test the relationship between CRF and EFs, we must first verify that our two levels of complexity induced different demands. Thus, the first secondary objective was to test the effect of complexity on executive performance and perceived difficulty which is the subjective perception of the participants regarding the two levels of complexity. The second secondary goal was to follow the evolution of cardiorespiratory data to identify the peak of oxygen uptake (VO_2_ peak). Based on previous results, we expected a deterioration of executive performance and an increase in perceived difficulty with the increasing complexity of the executive task, which would reflect that the two levels of complexity induce different demands. Finally, CRF will correlate positively with executive performance, especially in the most complex condition.

## Materials and methods

### Study population

Twenty-nine young adults including 12 women, aged 18–25 years (20.6 ± 1.8 years) were enrolled in the study. Other participants’ characteristics were weight = 67.5 ± 9.7 kg; height = 1.73 ± 0.08 m; body mass index = 22.3 ± 1.9 kg m^–2^; education level = 14.6 ± 1.1 years. None of them suffered from cardiovascular, neurological, or rheumatoid diseases and had a medical certificate of aptitude to perform a submaximal effort test. Almost all participants were right-handed according to the Edinburgh laterality inventory (Oldfield, 1971) except two left-handed participants. Participants who could not have all the data of interest during the experimental session were excluded from the analysis. All participants signed an informed consent form before taking part in the experimental session, and the study was approved by the local ethics committee of the Université Paris Cité (no. IRB:00012020-111) in accordance with the ethical standards defined in the declaration of Helsinki.

### Assessment of inhibition and updating of working memory

To measure inhibition and updating of working memory, the Random Number Generation (RNG) task was used (Towse and Valentine, 1997; Towse, 1998; Albinet et al., 2006), where in participants were requested to say aloud a number from one to nine at a constant rate with an instruction that the string of numbers is supposed to be in an order as random as possible (Audiffren et al., 2009). The various numbers given by the participants during the task were recorded with a Dictaphone (Philips DVT 4110). Two levels of cognitive loads of this task were implemented: regarding the low level of cognitive load (RNG1), participants were requested to say a random number every 1.5 s; in the case of the high level of cognitive load (RNG2), participants were requested to produce a random number every single second. The RNG task involves inhibition as well as updating of working memory, which are two EFs (Miyake et al., 2000). Several indices calculated by the Rgcalc software, based on the numbers generated by the participants, allow the assessment of inhibition and working memory updating [28]. For inhibition, we have the adjacency score (A score), the Turning Point Index (TPI), and the Runs Score (RS). Regarding the updating of working memory, we have the Redundancy Index (R index), the Coupon Score (CS), and the Mean Repetition Gap (Mean RG).

According to Audiffren et al. (2009), A score measures—the percentage of adjacent ascending or descending number pairs relative to the total number of response pairs produced by the participant p. 87. —It ranges from 0 to 100. A low A score means better inhibition ability. Regarding the TPI, it is a—measure of the regularity of sequences that make changes from ascending to descending sequences p. 87. —The TPI ranges from 0 to 150 and, when it is higher than 100, it reflects better inhibition ability. The last index of inhibition is the RS, which is -the number of items in successive ascending sequences p. 87. —The RS ranges from 0 to 6.82, and a low RS reflects better inhibition ability. Like inhibition, three indices can be used to assess updating of working memory. Among these indices, we have R index, which represents—the ratio between the quantity of information provided by the sequence of ciphers generated by the participant and the maximum amount of information that can be generated in such a sequence p. 87. —It varies between 0 and 100%, and a low R index reflects a high updating ability. Besides the R index, there is a second index that the CS, which represents the—number of ciphers said by the participant before all the ciphers alternatives have been given p. 87. —It varies between 9 and 100, and a low CS reflects a high updating ability. Finally, there is the Mean RG, -which is the average number of ciphers between successive occurrences of the same ciphers for all ciphers in the whole sequence p. 87. —It varies between 1 and 9, and a high Mean RG reflects a better updating ability.

### Assessment of cardiorespiratory fitness

The RABIT^®^ is self-paced determination test used to assess the CRF determined by the VO_2_ peak. This test has recently been developed, and cardiorespiratory parameters measured during this last were well related with parameters measured during the graded exercise test (*r*^2^ = 0.83) (Giovanelli et al., 2020) and the University of Montreal track test (*r*^2^ = 0.79) (Molinari et al., 2020). More precisely, the RABIT^®^ test is validated to measure the maximal oxygen consumption obtained because its values of maximal oxygen consumption are correlated with the values obtained with the University of Montreal track test. In addition, there is no significant difference between maximal parameters (e.g., VO_2_ max, maximal heart rate, maximal respiratory exchange ratio) measured with the RABIT test and with a graded exercise test (i.e., incremental exercise) (Giovanelli et al., 2020). Moreover, the cardiorespiratory responses assessed with the RABIT^®^ test are reliable as a function of the running intensity (Molinari et al., 2020). Thus, the peak of VO_2_ is reached close to maximal intensity of exercise during the test.

This test involves four steps during which the following instructions are given to participants: (a) run 10 min at a free warm-up pace, (b) run 5 min at a moderate pace, (c) run 3 min at a hard pace, (d) run 10 min at an easy pace (Giovanelli et al., 2020). Between steps (a) and (b), a participant walks for 25 s, sprints for 10 s, and walks again for 25 s. In contrast, between steps (b) and (c), a participant walks for 1 min. Finally, between steps (c) and (d) a participant walks for 15 s, then does a 30-s sprint, and walks again for 15 s. During the test, participants were equipped with a respiratory gas analyzer (Metamax 3B-R2) and a heart rate monitor belt (Polar) to collect cardiorespiratory data VO_2_ peak, heart rate, ventilatory equivalent (VE/VO_2_), minute ventilation/carbon dioxide production (VE/VCO_2_), respiratory exchange ratio (RER); peak ventilation (VE peak). The effect of the weight of the participants on their VO_2_ peak was corrected using the allometric scaling method which consists of raising the weight to the power of 0.75 before relating it to the VO_2_ peak (Bergh et al., 1991).

### Experimental protocol

The experimental session comprised two stages. In the first stage, participants completed the Edinburgh laterality inventory (Oldfield, 1971) while specifying their age, gender, weight, height, and education level. Next, participants performed the two levels of cognitive loads of the RNG task (RNG1 and RNG2), which last 100 and 150 s, respectively. Thus, at the end of each level of complexity, a participant must give 100 digits. To avoid an order effect, the two levels of cognitive loads were counterbalanced between the two participants. Before completing each level of complexity, participants did a 30-s familiarization phase. If the experimenter feels that a participant has not fully under-stood the task, he asks the participant to resume the familiarization phase. At the end of each level of complexity, participants rated the perceived difficulty using the 6–20 Borg scale (Borg, 1970), which was adapted by asking them to specify their perception of the ‘mental effort’ they had made to complete the task. The second phase was devoted to the effort test. Before the test, we have equipped a participant with the respiratory gas analyzer and the heart rate monitor. During the test, 10 s before the end of each step (a, b, c, and d see section Materials and methods), we asked the participants about their perception of effort using the 6–20 Borg scale (Borg, 1970).

### Statistical analysis

All statistical analyses were conducted in JASP 0.14.1^1^. We first tested the normality of our data using the Shapiro–Wilk test. To test the effect of complexity on participants’ executive performance, perceived difficulty, and effect of gender, we used the student’s test on A score, TPI, RS, R index. On the other hand, as the CS, Mean RS, and perceived difficulty are not normally distributed, we have used the Wilcoxon rank test or Mann-Whitney.

We then performed a repeated measures Anova (step a vs. b vs. c vs. d) on cardiorespiratory data of all participants as VO_2_ peak, heart rate, RER, Ve/VO_2_, Ve/VCO_2_, Ve’max, after checking the sphericity with the Mauchly sphericity test. When the assumption of sphericity is violated, a Greenhouse-Geisser correction is performed. Whenever necessary, we applied Bonferroni corrections to explore multiple comparisons. Regarding the perceived effort, we performed the Friedman test because these data were not normally distributed. Multiple comparisons were performed with the Conover test. Finally, a comparison between women and men was also carried out on each cardiorespiratory data using the student’s *t*-test or the Mann–Whitney test.

Finally, to test the relationship between the executive performance, VO_2_ peak, and perceived difficulty of RNG, we ran Pearson’s correlations. This analysis was carried out on all participants but also according to gender. For all these analyses, the level of significance was set at *p* ≤ 0.05, and we reported the effect sizes (Cohen’s d or η2).

## Results

During the experimental protocol, four participants (one woman and three men) were unable to complete the RABIT^®^ test due to technical failure of the respiratory gas analyzer. The results presented in this section consider 25 participants. On a mean, participants gave (99.1 ± 2.9) and (96.8) digits during RNG1 and RNG2, respectively.

### Executive performances and perceived difficulty

#### Inhibition

The student test showed an effect of complexity on A score, *t* (24) = −4.7; *p* < 0.001; Cohen’s *d* = 0.9, TPI, *t* (24) = 2.8; *p* = 0.009; Cohen’s *d* = 0.6, RS, *t* (24) = −2.4; *p* = 0.02; Cohen’s *d* = 0.5. This result indicates deterioration in inhibition capacity during RNG2 compared to RNG1 for all inhibition indices used. This deterioration is reflected by increase in the A score, RS, and a decrease of the TPI during RNG2 (Table 1). Finally, women and men do not differ on inhibition regardless of the inhibition index (*p* > 0.05).

**TABLE 1:** Changes in executive performances as a function of difficulty and gender.

	Women		Men		All groups	
	RNG1	RNG2	RNG1	RNG2	RNG1	RNG2
A score	29.32 ± 8.23	35.72 ± 10.89	27.41 ± 11.32	35.56 ± 13.13	28.25 ± 9.93	35.63 ± 11.95**
TPI	87.84 ± 14.72	79.57 ± 14.48	91.59 ± 12.31	85.12 ± 15.89	89.94 ± 13.27	82.68 ± 11.95**
RS	1.18 ± 0.62	1.49 ± 0.59	1.05 ± 0.37	1.40 ± 0.74	1.11 ± 0.49	1.44 ± 0.67**
R index	2.29 ± 1.53	2.56 ± 1.94	1.34 ± 0.88*	2.39 ± 1.50	1.76 ± 1.28	2.46 ± 1.67**
CS	18.96 ± 3.70	20.36 ± 8.32	17.50 ± 3.21	20.62 ± 7.80	18.14 ± 3.44	20.50 ± 7.86
Mean RS	7.18 ± 0.40	7.27 ± 0.78	7.75 ± 0.70*	7.28 ± 0.72	7.50 ± 0.64	7.28 ± 0.73

A score, Adjacency score; TPI, Turning Point Index; RS, Runs Score; R index, Redundancy index; CS, Coupon Score; Mean RS, Mean Repetition Gap. *Significant difference between women and men during RNG1, **Significant difference between RNG1 and RNG2.

#### Updating of working memory

The student test showed an effect of complexity on R index, *t* (24) = −2.5; *p* = 0.021; Cohen’s *d* = 0.5. In contrast, the Wilcoxon rank test performed on CS and Mean RG showed no significant effect of the complexity of these indices (*p* > 0.05). This result reveals deterioration in updating of working memory during RGN2 compared to RNG1. However, this is observed only at the level of R index. Finally, the Mann–Whitney test reveals that women and men differ significantly on two indices of updating of working memory, namely R index (*p* = 0.029) and Mean RG (*p* = 0.01) during RNG 1 (Table 1). This result indicates that men perform better on both indices than women.

#### Perceived difficulty

The Wilcoxon rank test performed on the perceived difficulty showed a significant effect of complexity (*p* = 0.015). Indeed, participants perceived RNG2 (12.5 ± 2.7) to be more challenging than RNG1 (11.2 ± 2.1). Finally, there was no gender effect on the perceived difficulty.

### Cardiorespiratory data and perceived effort

#### Cardiorespiratory data

The Greenhouse-Geisser correction performed on repeated measures Anova after the sphericity assumption is violated showed an effect of the step on VO_2_ peak, F (1.94, 46.68) = 55.78; *p* < 0.001, η^2^ = 0.69, indicating that VO_2_ peak increased between steps (a) and (c) before decreasing at step (d) (Table 2). Contrast analysis showed that there is a difference between step (a) and step (b) *t* (72) = −8.94; *p* < 0.001 and between step (c) and (d) *t* (72) = 8.92; *p* < 0.001. However, there was no significant difference between steps (b) and (c) *t* (72) = −1.94; *p* = 0.055. The same analyses performed on Heart rate showed an effect of the step on Heart rate, *F* (2.24, 53.76) = 60.4; *p* < 0.001, η^2^ = 0.71, indicating that Heart rate increased between steps (a) and (c) before decreasing at step (d) (Table 2). Contrast analysis revealed significant differences between steps (a) and (b) *t* (72) = −9.49; *p* < 0.001; between steps (b) and (c) *t* (72) = −2.74; *p* = 0.008; and between steps (c) and (d) *t* (72) = 8.29; *p* < 0.001. Regarding VE/VO_2_, the repeated measures Anova also showed a significant effect of the step, *F* (3, 72) = 38.9; *p* < 0.001, η^2^ = 0.61, revealing that VE/VO_2_ increased between steps (a) and (c) before decreasing at step (d) (Table 2). Contrast analysis revealed significant differences between steps (a) and (b) *t* (72) = −6.35; *p* < 0.001; between steps (b) and (c) *t* (72) = −4.38; *p* < 0.001; and between steps (c) and (d) *t* (72) = 4.76; *p* < 0.001. The same analysis carried out on VE/VCO_2_ showed the same results *F* (3, 72) = 42.57; *p* < 0.001, η^2^ = 0.63 (Table 2). Contrast analysis showed significant differences between steps (a) and (b) *t* (72) = −4.74; *p* < 0.001; between steps (b) and (c) *t* (72) = −4.26; *p* < 0.001. However, there is no difference between steps (c) and (d) (*p* = 0.25). The Greenhouse-Geisser correction performed on repeated measures Anova after the sphericity assumption was violated showed an effect of the step on RER, *F* (2.21, 53.05) = 66.02; *p* < 0.001, η^2^ = 0.73 (Table 2). Contrast analysis indicated significant differences between steps (a) and steps (b) *t* (72) = −7.4; *p* < 0.001; between steps (b) and (c) *t* (72) = −3.16; *p* = 0.002; and between steps (c) and (d) *t* (72) = 11.9; *p* < 0.001. Finally, repeated measures Anova performed on VE peak showed a significant effect of the step, *F* (3, 72) = 58.5; *p* < 0.001, η^2^ = 0.7 (Table 2). Contrast analysis showed significant differences between steps (a) and (b) *t* (72) = −8.6; *p* < 0.001; between steps (b) and (c) *t* (72) = −3.76; *p* < 0.001; and finally, between steps (c) and (d) *t* (72) = 8.51; *p* < 0.001.

**TABLE 2:** Evolution of cardiorespiratory parameters according to the steps of VO_2_max test and gender.

	Steps of VO_2_max test											
	Women				Men				All groups			
	(a)	(b)	(c)	(d)	(a)	(b)	(c)	(d)	(a)	(b)	(c)	(d)
VO_2_ peak (mL.min^–1^.kg BW^–0.75^)	87.57 ± 14.0	106.92 ± 11.33	108.40 ± 13.16	89.37 ± 14.23	101.99 ± 13.01*	134.44 ± 15.97*	143.21 ± 16.30*	110.90 ± 12.43*	95.65 ± 15.07	122.12 ± 19.81^‡^	127.89 ± 22.96**	101.48 ± 16.97^†^
Heart rate (bpm)	164.44 ± 18.78	180.92 ± 13.90	184.36 ± 13.76	169.59 ± 18.29	153.67 ± 13.01	175.07 ± 11.32	182.30 ± 10.41	163.88 ± 13.17	158.4 ± 16.38	177.6 ± 12.60^‡^	183.2 ± 11.82**	166.4 ± 15.55^†^
VE/VO_2_	26.91 ± 1.44	29.93 ± 2.64	32.79 ± 3.35	30.5228.75 ± 3.70	23.03 ± 2.03*	26.78 ± 3.47*	28.75 ± 3.70*	25.95 ± 2.56*	24.7 ± 2.64	28.2 ± 3.46^‡^	30.5 ± 4.04**	27.9 ± 4.10^†^
VE/VCO_2_	28.55 ± 1.86	30.00 ± 261	31.98 ± 2.78	32.58 ± 3.96	24.61 ± 2.15*	26.60 ± 3.10*	27.85 ± 3.32*	28.14 ± 2.96*	26.3 ± 2.82	28.1 ± 3.35^‡^	29.7 ± 3.68**	30.1 ± 4.04^†^
RER	0.94 ± 0.02	0.99 ± 0.03	1.02 ± 0.05	0.93 ± 0.02	0.93 ± 0.03	1.00 ± 0.04	1.03 ± 0.04	0.92 ± 0.03	0.94 ± 0.02	1.00 ± 0.04^‡^	1.03 ± 0.05**	0.92 ± 0.02^†^
VE peak (L/s)	3.06 ± 0.71	4.14 ± 0.82	4.41 ± 0.85	3.42 ± 0.83	3.53 ± 0.57	5.17 ± 1.05	6.04 ± 0.82*	4.37 ± 063*	3.3 ± 0.66	4.7 ± 1.07^‡^	5.3 ± 1.16**	3.9 ± 0.85^†^

(a) Run 10 min at a free warm-up pace; (b) run 5 min at a moderate pace; (c) run 3 minutes at a hard pace; (d) run 10 min at an easy pace; VE/VO2, ventilatory equivalent; VE/VCO2, minute ventilation/carbon dioxide production; RER, respiratory exchange ratio; VE peak, peak ventilation. *Significant difference between women and men, ^‡^Significant difference between (a) and (b), **Significant difference between (b) and (c), ^†^Significant difference between (c) and (d).

The comparison between women and men showed that at all steps of the RABIT^®^ test there was a significant difference between women and men on VO_2_ peak: (a) *t* (23) = −2.65; *p* = 0.014; (b) *t* (23) = −4.90; *p* < 0.001; (c) *t* (23) = −5.75; *p* < 0.001; (d) *t* (23) = −4.05; *p* < 0.001, VE/VO_2:_ (a) *t* (23) = 5.33; *p* < 0.001; (b) *t* (23) = 2.48; *p* = 0.02; (c) *t* (23) = 2.82; *p* = 0.01; (d) *t* (23) = 3.27; *p* = 0.003, VE/VCO_2:_ (a) *t* (23) = 4.80; *p* < 0.001; (b) *t* (23) = 2.87; *p* = 0.009; (c) *t* (23) = 3.31; *p* = 0.003; (d) *t* (23) = 3.20; *p* = 0.004. However, there was no difference in heart rate and RER. Finally, men and women differ significantly on VE peak in steps (c): *t* (23) = −4.84; *p* < 0.001 and (d): *t* (23) = −3.24; *p* = 0.004 (Table 2).

#### Perceived effort

Friedman’s Anova performed on perceived effort during the RABIT^®^ test showed a step effect (Chi-2 = 67.18; *df* = 3; *p* < 0.001). Indeed, the perceived effort increases from 8.04 ± 1.69 (a) to 12.28 ± 2.32 during step (b) and to 16.4 ± 1.68 during step (c). Finally, this perceived effort decreases to 9.4 ± 2.34 during step (d). Conover’s *post-hoc* comparisons showed significant differences between steps (a) and (b) (*p* < 0.001); between steps (b) and (c) (*p* = 0.012); and between steps (c) and (d) (*p* < 0.001). Finally, there is no significant difference between men and women in terms of perceived effort.

Findings of the RABIT^®^ test show that, although there is no significant difference between the VO_2_ of steps (b) and (c), only step (c) has an RER > 1.01, a heart rate > 180, and an average perceived effort of 16.4, which corresponds to arduous effort according to the Borg, 1970 scale. Thus, the VO_2_ in step (c) was used as the VO_2_ peak to explore the relationship between CRF, EFs and perceived exertion.

### Relationship between cardiorespiratory fitness and executive functions

To test the correlation between CRF and EFs at each level of complexity measured during our study, we first performed a Pearson correlation. Then, we performed the same analysis using executive performance as the difference between RNG2 and RNG1 for each index of inhibition and updating of working memory. Contrary to our expectations, there was no relationship between CRF and EFs (inhibition or updating of working memory) when all participants are included in the analysis with no distinction of gender. The same results are observed for women. In contrast, for men, Pearson correlation showed that the increase of VO_2_ peak is accompanied by the decrease of TPI (*r* = −0.67; *p* = 0.008; Upper 95% = −0.22 and lower 95% = −0.88) and the increase of R index (*r* = 0.66; *p* = 0.009; Upper 95% = 0.88 and lower 95% = 0.20) during RNG1. These results indicate that poorer executive performance during RNG1 is observed in men with high VO_2_ peak.

## Discussion

In the present study, we aimed to test the relationship between the directly assessed CRF and EFs in young adults while considering the level of mental workload. To test this relationship, it was necessary to examine the effect of complexity on executive performance and perceived difficulty and to explore the evolution of cardiorespiratory data during the RABIT^®^ test.

The present study showed the effect of complexity on perceived difficulty and executive performances. Indeed, when moving from RNG1 to RNG2 by increasing the rate of digit generation, participants perceived the task as more difficult and their inhibition and updating abilities decreased. However, while all three indices of inhibition were sensitive to complexity (i.e., A Score, TPI, RS), only one index of updating was sensitive to complexity (i.e., R index). The deterioration in performance during RNG2 compared to RNG1 was in line with previous studies (Towse, 1998; Jahanshahi et al., 2006). According to Jahanshahi et al. (2006), this deterioration can be explained by a high level of frequency of number generation. The participants’ executive central is unable to actively inhibit the default counting bias because executive resources are limited to manage the increase in the number generation frequency. Additionally, a lack of inhibition will lead participants to adopt a counting strategy and thus repeat the same digits, which would increase the R index. We also observed a difference between men and women on some indices of working memory updating (R index and Mean RG). This result deserves to be further investigated in future studies, because so far, no study using this task has tested the effect of gender.

The present study also reported that the RABIT^®^ test showed the highest VO_2_, heart rate, and RER during step (c), when participants were asked to run at a sustained intensity. Moreover, the perceived exertion of participants reached an average of 16.4 ± 1.7, which reflected an intense perception of effort. This result is in line with previous findings (Giovanelli et al., 2020; Molinari et al., 2020) demonstrating that the RABIT^®^ test is a pertinent test to assess submaximal and maximal parameters of CRF.

Finally, the relationship between CRF and EFs did not reach statistical significance in our population of young active adults. This result is consistent with the study of Hayes et al. (2016), who pointed out the lack of relationship between CRF and EFs in young adults (Hayes et al., 2016) and in adolescents (Cabral et al., 2021). On the contrary, studies performed with older subjects highlighted this relationship between CRF and EFs (Kawagoe et al., 2017; Mekari et al., 2019). Indeed, physical fitness may be beneficial in maintaining executive functions in healthy aging by enhancing the efficiency of the global brain network (Kawagoe et al., 2017) and cerebral oxygenation (Mekari et al., 2019). These differences could be explained by the ability of young adults to use more their frontal cortex during EFs which strengthens its structural connectivity (Sousa et al., 2018) and allows them to be at the peak of their executive performance (Hötting and Röder, 2013; Hayes et al., 2016). Thus, it is hypothesized that the demands induced by the two levels of complexity were not beyond the resources of the young adults included in our study. Perhaps by proposing a third level of complexity that implies more requirements we could have highlighted the relationship between CRF and EFs in this specific population. However, these results should be taken with caution because when we tested the relationship between CRF and executive performance by taking gender into account, we found that there was a relationship between CRF and executive performance only in men. Future studies should perhaps be conducted on a single gender (male or female).

One of the study limitations was the small sample size. However, the study was conducted during the COVID-19 pandemic that did not allow us to recruit many participants. This small sample size precludes high statistical power, which may explain the lack of relationship between CRF and executive performances. The use of a larger sample size is therefore essential and to achieve this, researchers need to standardize their experimental protocol to perform a multi-laboratory study (Ciria et al., 2022). Another limitation was that we measured only two of the three core EFs. It would be therefore interesting to consider cognitive flexibility but also higher-order EFs in the investigation of correlations between executive control and CRF. At the same time, it will also be required to measure cerebral hemodynamic activity using near-infrared spectroscopy (Pinti et al., 2020) to examine an indirect effect of CRF on EFs through brain activity. Several executive functions will be measured to determine a composite performance that will be related to the hemodynamic activity of the prefrontal cortex as a function of CRF. Future studies examining these different issues may be conducted in healthy young adults and their peers with cardiovascular disease. Indeed, previous studies have showed that there is an impairment of executive functions in patients with heart disease (Eggermont et al., 2012; Calderon and Bellinger, 2015). Thus, showing a correlation between CRF and EFs in this population could be a way to improve the care and solve the cognitive problems of young adults with cardiovascular abnormalities (e.g., Marfan syndrome) or patients with congenital cardiac defects. These studies are all the more important in view of the lack of data on this issue in young adults and the increase in physical inactivity and sedentary behavior among them. In addition, since the CRF and EFs relationships is partly dependent on age, future studies in this field should enroll subjects with different age categories (i.e., 5–11; 12–17; 18–30; 31–40; 41–50; 51–60 and >60 years old) to detect when there is a relationship and try to explain precisely why.

In sum, the results of this study were in line with previous research showing a lack of correlation between CRF and EFs in this specific population. In addition, it was demonstrated the effect of complexity on participants’ executive performance and the perceived difficulty. Additional studies are needed to further explore the relationship between CRF and EFs in healthy young adults and those with cardiovascular disease and in subjects with different age categories.

## Data availability statement

The raw data supporting the conclusions of this article will be made available by the authors, without undue reservation.

## Ethics statement

The studies involving human participants were reviewed and approved by local ethics committee of the Université Paris Cité (no. IRB:00012020-111). The patients/participants provided their written informed consent to participate in this study.

## Author contributions

NA and DV contributed to conception study. MPM and NA collected data. NA, PM, and DV processed. NA, MPM, PM, and DV wrote sections of the manuscript. All authors contributed to manuscript revision, read, and approved the submitted version.

## Conflict of interest

The authors declare that the research was conducted in the absence of any commercial or financial relationships that could be construed as a potential conflict of interest.

## Publisher’s note

All claims expressed in this article are solely those of the authors and do not necessarily represent those of their affiliated organizations, or those of the publisher, the editors and the reviewers. Any product that may be evaluated in this article, or claim that may be made by its manufacturer, is not guaranteed or endorsed by the publisher.

## Footnotes

1
https://jasp-stats.org/
